# Complete Chloroplast Genome Sequence Structure and Phylogenetic Analysis of Kohlrabi (*Brassica oleracea* var. *gongylodes* L.)

**DOI:** 10.3390/genes15050550

**Published:** 2024-04-26

**Authors:** Mengliang Zhao, Yanxun Wu, Yanjing Ren

**Affiliations:** 1 State Key Laboratory of Plateau Ecology and Agriculture, Qinghai University, Xining 810016, China; 8304269@163.com; 2 Qinghai Academy of Agriculture and Forestry Sciences, Xining 810016, China; wuyanxun1001@163.com; 3 Laboratory of Research and Utilization of Germplasm Resources in Qinghai-Tibet Plateau, Xining 810016, China; 4 Qinghai Provincial Key Laboratory of Vegetable Genetics and Physiology, Xining 810016, China

**Keywords:** kohlrabi, chloroplast genome sequence structure, gene annotation, relative synonymous codon usage, interspersed repeat sequence analysis, phylogenetic tree

## Abstract

Kohlrabi is an important swollen-stem cabbage variety belonging to the Brassicaceae family. However, few complete chloroplast genome sequences of this genus have been reported. Here, a complete chloroplast genome with a quadripartite cycle of 153,364 bp was obtained. A total of 132 genes were identified, including 87 protein-coding genes, 37 transfer RNA genes and eight ribosomal RNA genes. The base composition analysis showed that the overall GC content was 36.36% of the complete chloroplast genome sequence. Relative synonymous codon usage frequency (RSCU) analysis showed that most codons with values greater than 1 ended with A or U, while most codons with values less than 1 ended with C or G. Thirty-five scattered repeats were identified and most of them were distributed in the large single-copy (LSC) region. A total of 290 simple sequence repeats (SSRs) were found and 188 of them were distributed in the LSC region. Phylogenetic relationship analysis showed that five *Brassica oleracea* subspecies were clustered into one group and the kohlrabi chloroplast genome was closely related to that of *B. oleracea* var. *botrytis.* Our results provide a basis for understanding chloroplast-dependent metabolic studies and provide new insight for understanding the polyploidization of Brassicaceae species.

## 1. Introduction

Kohlrabi (*Brassica oleracea* var. *gongylodes* Linnaeus, 1753), an important swollen-stem vegetable variety of *B. oleracea* variety, originated from northwestern Europe and is widely cultivated in Europe, the US, Canada, and Asia [1]. The swollen stem at the base of the plant is mainly consumed by humans as food [2,3]. Studies have shown that the swollen stem of kohlrabi has high nutritional value, particularly in vitamin C, vitamin E, and tocopherols [4,5,6]. In addition, potential antidiabetic, anti-inflammatory, and antioxidant properties and anticancer effects have been found in kohlrabi [7].

Chloroplasts (cp) are centers of plenty cellular reactions and crucial organelles of plant cells [8,9], originating from photosynthetic cyanobacteria engulfed and enslaved by eukaryotic cells [10,11]. Chloroplasts not only play vital roles in photosynthesis but also contain all the elements required for carbohydrate metabolism and biosynthesis of nucleotides and amino acids [12]. In addition, chloroplasts are involved in various molecular processes, such as regulation of plant physiology, growth, development, and stress responses [12,13,14].

The cp genome is a maternally inherited genetic systems of plants, which is not affected by karyogene deletion, overlap, or pseudogenes [15]. In the cp genome, a large number of mutational events also occur, such as nucleotide substitutions, insertions, deletions, and genome fragment inversions, translocations and rearrangements [16,17,18]. In most angiosperms, chloroplasts have a typical quadripartite circular genome, which comprises one large single-copy (LSC) region with a length of about 81–90 kb, one small single-copy (SSC) region of approximately 18–20 kb, and two inverted repeat (IR) regions of about 20–30 kb, named IRa and IRa [13,15,19]. Oldenburg and Bendich [20] reported that a linear cp genome was found in maize. As plant-specific organelles, chloroplasts have highly conserved genomes in terms of gene content and organization while maintaining a relatively simple structure, small molecular weight and large copy number [21,22,23], owing to a high conservation of structure, moderate genome size and good collinearity between various plant groups of cp genomes [24], which have been widely used in DNA fingerprint development, phylogenetic discrepancy analysis, molecular evolution, and genetic engineering modification, such as *B. rapa*. ssp. *rapa* [25], *Paeonia ostii* [26], *Zingiber officinale* [27], *Withania somnifera* [28], *Adrinandra megaphylla* Hu [29], and *Cornus* species [30]. Moreover, cp genomes have also been used to deal with important scientific issues of crop origin and domestication [31].

Several cp genome sequence have been reported in *B. oleracea*: *B. oleracea* var. *botrytis* (cauliflower) (KX681655.1), *B. oleracea* var. *italica* (cabbage) (MH388765, MN649876.1), *B. oleracea* var. *capitata* (red cabbage) (KR233156.1), *B. oleracea* (wild cabbage) (MG717288) and *B*. *oleracea* var. *alboglabra* (white-flower Chinese kale and yellow-flower Chinese kale) (OR063915 and OR063916). However, despite being the only swollen-stem vegetable of the *B. oleracea* variety, no sequence of the kohlrabi cp genome has been presented to our knowledge. Thus, the complete sequence of the kohlrabi cp genome was obtained and analyzed in this study.

## 2. Materials and Methods

### 2.1. Plant Materials and DNA Extraction

**Figure 1:**
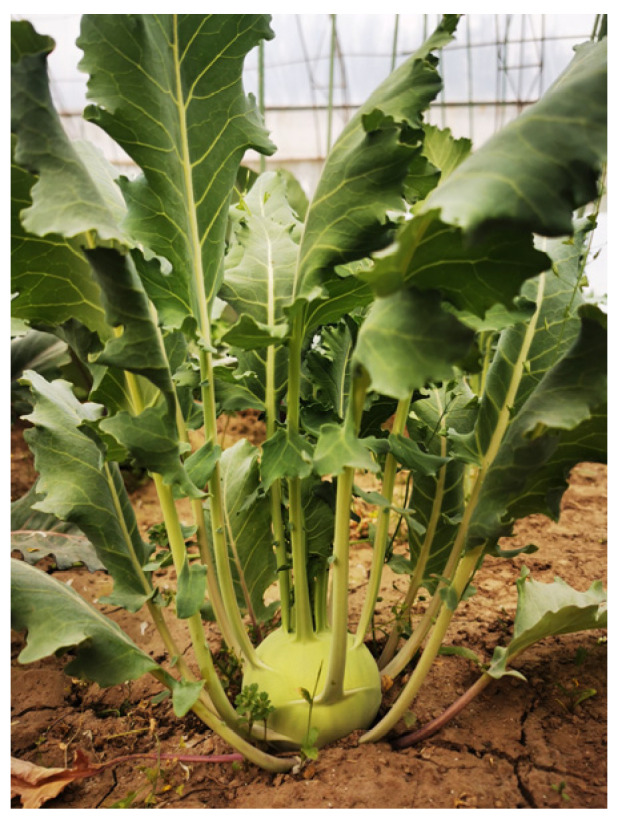
The kohlrabi plant grown in the field and used in this paper.

Kohlrabi seeds were sown in holed plates in late April and cultured in greenhouse for 35 days until the seedlings had 4 or 5 leaves, then the seedlings were transplanted in a randomized field plot with regular management in an experimental field (36°420 N; 101°450 E) of the Academy of Agriculture and Forestry Sciences, Qinghai University. The fresh young kohlrabi leaves were collected and the modified CTAB (cetyl trimethyl ammonium bromide) method as Porebski et al. [32] described was used for total DNA extraction. The same DNA samples of Shao et al. [33] were used in this study and stored at the Qinghai Key Laboratory of Vegetable Genetics and Physiology. The kohlrabi plant were showed in Figure 1 and used in this paper.

### 2.2. DNA Sequencing and Assembly

The extracted DNA were assigned for purification and assessment, and then qualifying DNA were used to build a library. This library was sequenced using the Illumina NovaSeq2500 platform (Shaanxi Breeding Biotechnologies Co., Ltd., Xi’an, China). Using SPAdes v3.10.1 [34] software, the cp genome sequence was assembled and did not depend on the reference genome. Then, gap repair in the scaffolds was performed using GapFiller v2.1.1 [35] until a complete pseudogenome was obtained. Finally, based on the structure of chloroplasts and rearrangement of the pseudo genome, a complete cp circular genome was obtained.

### 2.3. Gene Annotation

Based on the assembled sequences of relative species uploaded to the National Center for Biotechnology Information (NCBI), the online software BLAST v2.6 (https://blast.ncbi.nlm.nih.gov/Blast.cgi, accessed on 23 March 2023) was used for gene alignment. Two strategies were used to annotate the cp genome for improving annotation accuracy. Firstly, the prodigal v2.6.3 (https://www.github.com/hyattpd/Prodigal, accessed on 23 March 2023), hmmer v3.1b2 (http://www.hmmer.org/, accessed on 23 March 2023) and aragorn v1.2.38 (http://130.235.244.92/ARAGORN/, accessed on 23 March 2023) were separately used to annotate the coding sequence (CDS), predict the ribosomal RNA (rRNA) and transfer RNA (tRNA). Secondly, based on the cp genomes of closely related species published on NCBI, we obtained annotation information by comparing the assembled sequences using BLAST v2.6 DOGMA (http://dogma.ccbb.utexas.edu/, accessed on 23 March 2023) [36]. Results from two strategies were manually checked for differentially annotated genes. Then, we removed any misannotation and redundant annotation and determined the multi-exon boundaries. Finally, the final annotation information was obtained. The tRNAs were analyzed based on the online software tool tRNAscan-SE with default settings (http://lowelab.ucsc.edu/tRNAscan-SE/, accessed on 23 March 2023) [37]. OrganellarGenomeDRAW (http://ogdraw.mpimp-golm.mpg.de/index.shtml, accessed on 23 March 2023) was applied for visualization of the complete cp genome map [38].

### 2.4. Codon Usage Frequency Analysis

According to the degeneracy of codons, each amino acid was coded by 1 codon at least and 6 codons at most. In different species and different organisms, there are great differences in genome codon usage rates. The inequality of synonymous codon usage is called relative synonymous codon usage (RESU). RESU was calculated using the following formula: RESU = ratio of (number of one of the codons encoding a certain amino acid) to (number of all codons encoding this amino acid)/(1/the codon species encoding this amino acid).

### 2.5. Repeat Sequence Analysis

The interspersed repeat sequence was searched using vmatch v2.3.0 (http://www.vmatch.de/, accessed on 23 March 2023) software, in which parameters were set as minimum length = 30 bp and Hamming distance = 3. Four forms of interspersed repeat sequences were identified: forward, palindromic, reverse and complementary repeat sequences. The SSRs were identified using MISA v1.0 (MIcroSAtellite identification tool, http://pgrc.ipk-gatersleben.de/misa/misa.html, accessed on 23 March 2023) software, in which parameters were set as single-base repeat > 8, di-base repeat > 5, tri-base repeat > 3, tetra-base repeat > 3, penta-base repeat > 3, and hexa-base repeat > 3 [15,39,40]. The SSR primers were designed using the SSR analysis results.

### 2.6. Cp Genome Comparison of Cabbage Species

Four variants of *B. oleracea* were selected for comparing the boundaries between the LSC, IR and SSC regions in the kohlrabi cp genome, including the newly assembled cp genome of *B. oleracea* var. *gongylodes* (MW900251, 153,364 bp), *B. oleracea* var. *alboglabra* cv. SJCT (OR063915, 153,365 bp) [15], *B. oleracea* var. *alboglabra* cv. FZHH (OR063916, 153,420 bp) [15] and *B. oleracea* var. *itaica* (MN649876.1, 153,364 bp) [23]. Boundary differences in IRB-LSC, IRB-SSC, IRA-SSC, and IRA-LSC were compared among these four variants with the annotation information of cp genomes available in GenBank.

### 2.7. Phylogenetic Analysis

Phylogenetic relationships were analyzed using MEGA7 by the maximum likelihood (ML) method [41]. The cp genomes of other species for phylogenetic relationships analysis were downloaded from the NCBI database, including *B. oleracea* (wild cabbage) (MG717288), *B. oleracea* var. *capitata* (KR233156.1), *B. oleracea* var. *botrytis* (KX681655.1), *B. oleracea* var. *italica* (MH388765), *B. juncea* (KT581449.1), *B. rapa* (NC_040849.1), *B. rapa* ssp. *rapa* (MT409177), *B. napus* (GQ861354.1), *B. nigra* (KT878383.1), *Raphanus staivus* (NC_024469.1), *Arabidopsis thaliana* (NC_000932.1), *Solanum lycopersicum* (NC_007898.3) and *Oryza sativa* (NC_031333.1). Of them, the cp genome sequence of *O. sativa* (NC 031333.1) was used as the outgroup.

## 3. Results

### 3.1. Complete Chloroplast Genome Assembly and Gene Annotation

**Figure 2:**
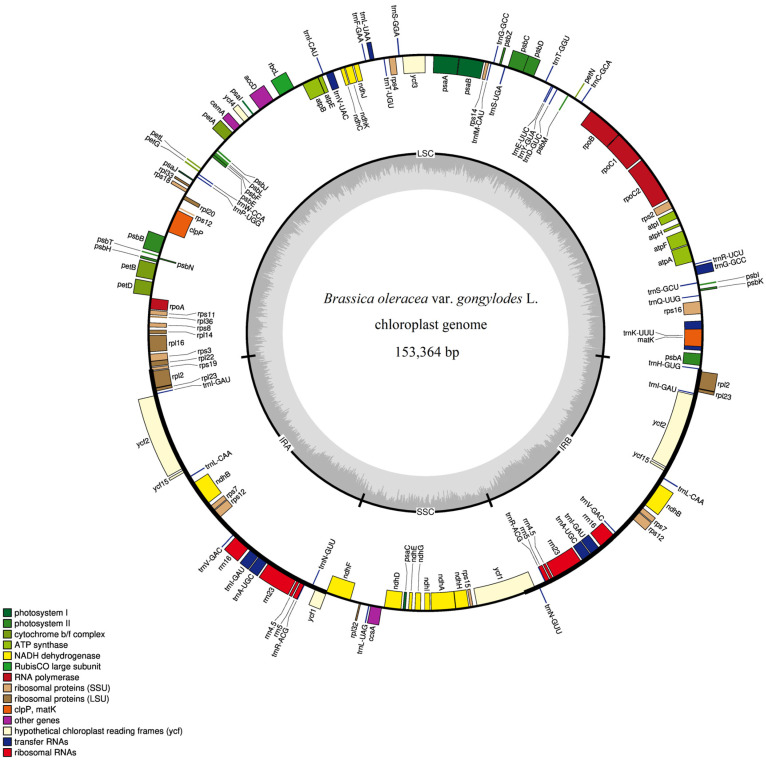
Quadripartite circular cp genome map of complete kohlrabi chloroplast genome. Note: Genes inside the circle are transcribed clockwise, and genes outside the circle are counterclockwise. The darker gray area and the lighter gray area in the inner circle correspond to GC content and AT content, respectively.

Based on the assembled sequences, the complete quadripartite circular cp genome of kohlrabi with a length of 153,364 bp without any gaps was generated. Similarly to cp genomes of other crops, there are four sequence regions of the kohlrabi cp genome, including a large single-copy region (LSC) with length of 83,136 bp, a small single-copy region (SSC) with length of 17,834 bp and two inverted repeats (IRa and IRb) with length of 26,197 bp. The base composition analysis showed that the overall GC and AT content was 36.36% and 63.64% of the complete cp genome sequence, respectively. The GC and AT content was 34.15%, 65.85% in the LSC region, 29.10%, 70.90% in the SSC region, and 42.35%, 57.65% in the IR regions, respectively. Based on the gene annotation results, the cp genome of kohlrabi contained 132 genes, 87 of which were annotated as protein-coding genes, 37 as tRNA genes, and 8 as rRNA genes. The complete cp genome map is shown in Figure 2. All sequence information and gene annotation of the complete kohlrabi cp genome has been uploaded to the NCBI database under GenBank accession number MW900251.

According to the different biosynthetic pathways and various functions of these 132 genes, 45 of them were annotated as involved in photosynthesis pathways, 74 as self-replication genes, 8 considered conserved hypothetical chloroplast open reading frames, and 5 were annotated as other genes (Table 1). Among these 45 genes involved in photosynthesis, 5 genes were involved in subunits of photosystem I—*psaA*, *psaB*, *psaC*, *psaI* and *psaJ*; 15 in subunits of photosystem II—*psbA*, *psbB*, *psbC*, *psbD*, *psbE*, *psbF*, *psbH*, *psbI*, *psbJ*, *psbK*, *psbL*, *psbM*, *psbN*, *psbZ* and *psbT*; 6 in subunits of the cytochrome b/f complex—*petA*, *petB*, *petD*, *petG*, *petL* and *petN*; 1, *rbcL*, in a large subunit of rubisco; six in subunits of ATP synthase—*atpA*, *atpB*, *atpE*, *atpF*, *atpH* and *atpI*; and 12 in subunits of NADH dehydrogenase—*ndhA*, ndhB (×2), *ndhC*, *ndhD*, *ndhE*, *ndhF*, *ndhG*, *ndhH*, *ndhI*, *ndhJ* and *ndhK*. Five genes contained one intron (*petB*, *petD*, *atpF*, *ndhA and ndhB*).

Among these 74 genes annotated as self-replication genes, 8 were annotated as rRNA genes, comprising *rrn16* (×2), *rrn23* (×2), *rrn4.5* (×2), and *rrn5* (×2). Fourteen genes were annotated as involving the small ribosome subunit: *rps2*, *rps3*, *rps4*, *rps7* (×2), *rps8*, *rps11*, *rps12* (×2), *rps14*, *rps15*, *rps16*, *rps18* and *rps19*. Eleven genes were annotated as involving the large ribosome subunit: *rpl2* (×2), *rpl14*, *rpl16*, *rpl20*, *rpl22*, *rpl23* (×2), *rpl32*, *rpl33*, and *rpl36*. Two genes contained one intron (*rps16* and *rpl2*) and one gene (*rps12*) contained two introns. Among 37 genes annotated as tRNA genes, seven occurred in two copies (*trnI-CAU*, *trnL-CAA*, *trnV-GAC*, *trnI-GAU*, *RNA-UGC*, *trnR-ACG* and *trnN-GUU*) and six contained one intron (*trnK-UUU*, *trnG-GCC*, *trnL-UAA*, *trnV-UAC*, *trnI-GAU* and *trnA-UGC*).

Five other genes were annotated involving maturase (*matK*), envelope membrane protein (*cemA*), subunit of acetyl-CoA (*accD*), c-type cytochrome synthesis gene (*ccsA*) and protease (*clpP*), respectively. Of these, *clpP* was found to contain two introns. Eight genes of unknown function were also identified: *ycf1* (×2), *ycf2* (×2), *ycf3*, *ycf4*, and *ycf15* (×2). Of these, *ycf3* was found to contain two introns.

Among 19 genes with two copies, except for *ycf1* residing within the SSC region and two copies of *rps12* residing within the LSC region and IR region, 17 were all located in the IR regions: *ndhB*, *rrn4.5*, *rrn5*, *rrn16*, *rrn23*, *rps7*, *rps12*, *rpl2*, *rpl23*, *trnI-CAU*, *trnV-GAC*, *trnL-CAA*, *trnI-GAU*, *trnA-UGC*, *trnR-ACG*, *trnN-GUU*, *ycf1*, *ycf2* and *ycf15*.

### 3.2. Relative Synonymous Codon Usage Analysis

**Figure 3:**
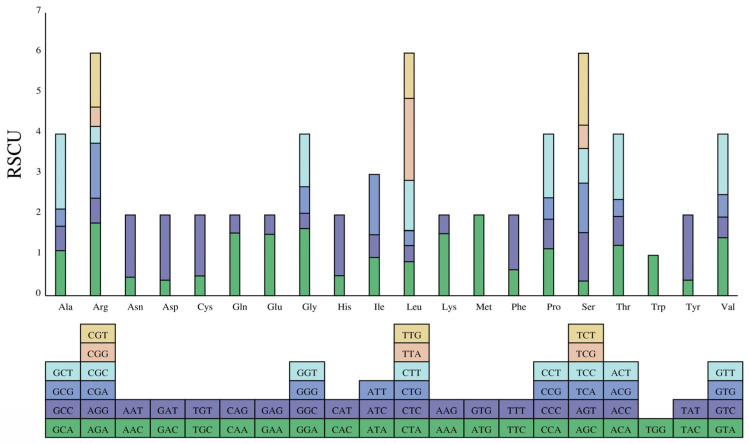
Analysis of codon preference in the kohlrabi chloroplast genome. Note: The colored blocks represent all codons encoding each amino acid, and the height of the upper column represents the sum of all codon RSCU values.

Based on the preference of codons used by CDS, we estimated the relative synonymous codon usage frequency (RSCU) and codon usage frequency. The codon–anticodon recognition patterns of the kohlrabi cp genome showed that a total of 30 tRNAs comprised codons corresponding to all 20 essential amino acids for protein biosynthesis. A total of 65 kinds of codons were searched in the cp genome, of which UAA had the highest usage encoding the termination codon. In addition, UUA for leucine, AUG for methionine, GCU for alanine, AGA for arginine, UCU for serine and GGA for glycine also had high usage (Figure 3, Table S1). Moreover, of all these 65 codons, 33 codons had RSCU values of >1, and 29 of them (93.50%) ended with base A or U, whereas the RSCU values for 31 codons were <1, and 30 of them (90.90%) ended with base C or G. Trp was encoded by only one UGG codon, indicating no biased usage (RSCU = 1).

### 3.3. Interspersed Repeat Sequence Analysis

**Figure 4:**
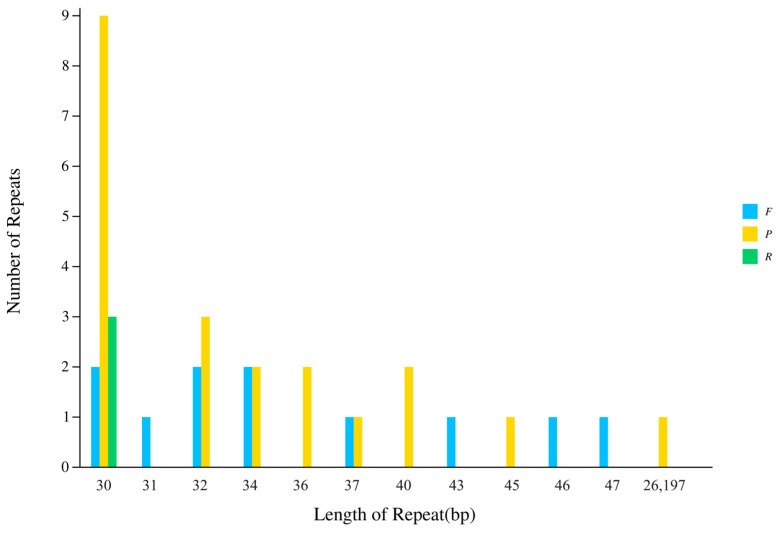
Summary of interspersed repeat sequence. F represents forward repeat, P represents palindrome repeat, R represents inverted repeat, and C represents complementary repeat.

Interspersed repeat sequence analysis identified a total of 35 scattered repeats: 11 forward, 21 palindromic, and 3 IRs (Figure 4). The positions of these interspersed repeat sequences were analyzed, and 21, 4, 2 and 7 were distributed in the LSC, SSC, IRa and IRb regions, respectively (Table S2). Repeat sequence lengths ranged from 30 to 47 bp, except the IR region.

### 3.4. Simple Sequence-Repeat Analysis

**Figure 5:**
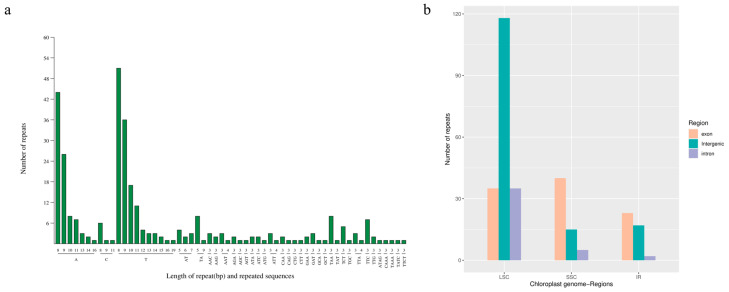
Summary and distribution of 290 simple sequence repeats. (**a**) SSR statistical data. (**b**) SSR distribution.

The SSR analysis revealed that 290 SSRs were identified, including 205 single-base, 18 di-base, 62 tri-base and five tetra-base repeats (Figure 5a). The positions of these SSRs were analyzed, and 188 (64.83%), 60 (20.69%) and 42 (14.48%) were distributed in LSC, SSC and IR regions, respectively (Figure 5b). A total of 98 (33.79%), 42 (14.48%) and 150 (51.72%) SSRs were located in exons, introns, and intergenic regions of the genome (Table S3). Based on the SSR analysis result, 283 pairs of primers were designed (Table S4).

### 3.5. Boundary Analysis

**Figure 6:**
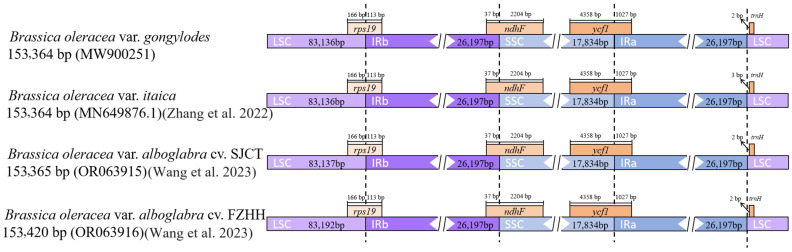
Comparison of gene location in LSC, IR, and SSC boundaries among four chloroplast genomes of *Brassica oleracea* varieties [15,23].

Four *B. oleracea* varieties were selected for boundary analysis between the LSC, IR and SSC regions in the cp genome: the newly assembled cp genome of *B. oleracea* var. *gongylodes* (MW900251, 153,364 bp), *B. oleracea* var. *alboglabra* cv. SJCT (OR063915, 153,365 bp) [15], *B. oleracea* var. *alboglabra* cv. FZHH (OR063916, 153,420 bp) [15] and *B. oleracea* var. *itaica* (MN649876.1, 153,364 bp) [23] (Figure 6). The lengths of the IR and SSC regions were the same in these four *B. oleracea* varieties. Only the length of LSC region was different, and ranged from 83,136 to 83,192 bp. The *rps19* coding sequence was located in the boundary of the LSC and IRb region and the relative location was same in these four *B. oleracea* varieties at 113 bp upstream of the IRb region. The *ndhF* and *ycf1* coding sequences were located in the boundaries of the IRb and SSC region and SSC and IRa region at 2204 bp upstream of the SSC region and 1027 bp upstream of the IRa region, respectively. The tRNA non-coding gene trnH-GUG in these four *B. oleracea* varieties was within the LSC region, which started 3 bp upstream of LSC in *B. oleracea* var. *gongylodes*, *B. oleracea* var. *alboglabra* cv. SJCT and *B. oleracea* var. *alboglabra* cv. FZHH, and 4 bp upstream of LSC in *B. oleracea* var. *itaica.* These results suggested that the boundaries between the LSC, IR and SSC regions were highly conserved except for minor differences in distance of trnH-GUG at the boundary between the IRa and LSC regions in *B. oleracea* varieties.

### 3.6. Phylogenetic Relationship Analysis

**Figure 7:**
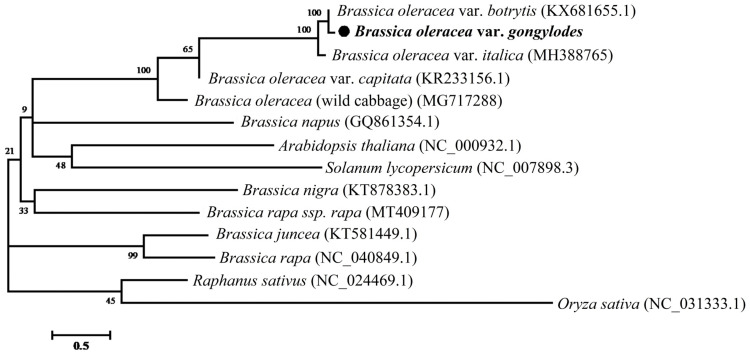
Phylogenetic relationships of thirteen species and kohlrabi based on the complete chloroplast genome sequence. The chloroplast sequence of *O. sativa* (NC 031333.1) was used as the outgroup. *B. oleracea* (wild cabbage) (MG717288), *B. oleracea* var. *capitata* (KR233156.1), *B. oleracea* var. *botrytis* (KX681655.1), *B. oleracea* var. *italica* (MH388765), *B. juncea* (KT581449.1), *B. rapa* (NC_040849.1), *B. rapa* ssp. rapa (MT409177), *B. napus* (GQ861354.1), *R. staivus* (NC_024469.1), *B. nigra* (KT878383.1), *A. thaliana* (NC_000932.1), *S. lycopersicum* (NC_007898.3) and *O. sativa* (NC_031333.1).

Phylogenetic relationships among eleven *Brassica* species, one *S. lycopersicum*, one *O. sativa*, and kohlrabi were determined by MEGA7 using the maximum likelihood (ML) method. Of these cp genome, *O. sativa* (NC_031333.1) was used as an outgroup (Figure 7). Five *B. oleracea* varieties were clustered into one group and three of them were clustered into one subgroup—*B. oleracea* var. *Botrytis* (KX681665.1)*, B. oleracea* var. *gongylodes* and *B. oleracea* var. *italica* (MH388765)—in which the kohlrabi cp genome was closely related to *B. oleracea* var. *botrytis*. These results may provide new insight for understanding the polyploidization between *Brassicaceae* species.

## 4. Discussion

Chloroplasts act as uniparentally inherited semi-autonomous organelles involved in the genetic systems of plants. Zhang et al. [23], Timmis et al. [41] and Liu et al. [42] reported that the gene number, gene composition, and gene arrangement of cp genomes are more highly conserved than those of mitochondrial and nuclear genomes. Based on the cp genome assembly, the length of the kohlrabi cp genome and GC content were similar to those of other Brassicaceae species, such as *B. oleracea* var. *alboglabra* [15], *B. oleracea* var. *italica* [23], B. *rapa* ssp. *rapa* [43], *R. sativus* L. [44], *B. nigra* and *B. oleracea* [45] and *B. juncea* (Indian mustard) [46]. The cp genome length of these species was approximately 153,300 bp to 153,500 bp and the GC content was approximately 36.30%. In terms of length of cp genome and GC content, they were highly conserved.

A total of 132 genes were identified and annotated in the cp genome of kohlrabi, fewer than that in *B. oleracea* var. *alboglabra* and *B. oleracea* var. *itaica.* Based on the gene annotation, 19 genes were identified with two copies in the IR regions. In addition, five *orf* genes of unknown function were also identified in the cp genome of kohlrabi, fewer than that in cpDNA of *A. thaliana* [47]. This result indicated that gene losses occurred in the cp genome of Brassicaceae family, which is similar to gene losses in the cp genome of other genera, such as *Asteraceae*, *Leguminosae* and *Gentianaceae* [48,49].

Codon usage frequency is a crucial factor influencing the evolution of the cp genome. According to the RSCU estimation, we found that most codons with RSCU values > 1 ended with A or U, while most codons with RSCU values < 1 ended with C or G. This result is consistent with *B. oleracea* var. *itaica* [23], *Magnoliz zenii* [50] and other species [51,52], suggesting that this phenomenon may be similar in plant cp genomes and codon usage frequency of the cp genome is also highly conserved.

In the cp genome of kohlrabi, 290 SSRs were identified, of which 205 (70.69%) belonged to single-base A or T repeats. The proportion of mononucleotide repeats among all SSRs of the kohlrabi cp genome was similar to that in *B. oleracea* var. *itaica* [23], *B. rapa* ssp. *rapa* [43], *Quercus acutissima* [53] and *Aristolochia* medicinal species [54]. Similarly to other reports, most identified SSRs were positioned at the intergenic region of the cp genome. A total of 283 pairs of primers designed relying on SSRs in the cp genome of kohlrabi could be used in DNA fingerprint development and phylogenetic discrepancy analysis.

## 5. Conclusions

The complete cp genome of kohlrabi with length of 153,364 bp without any gaps was sequenced and analyzed. In sum, 87 protein-coding genes, 37 tRNA genes, and 8 rRNA genes were annotated. The overall GC content was 36.36% of the complete cp genome sequence, and 35 scattered repeats and 290 SSRs were found and identified. Phylogenetic relationship analysis revealed that the kohlrabi chloroplast genome was closely related to that of *B. oleracea* var. *botrytis.* Our results provide a basis for understanding the chloroplast-dependent metabolic studies and provide new insights into the polyploidization of Brassicaceae species.

## Figures and Tables

**Table 1:** Structural characteristics of kohlrabi chloroplast genes.

Category for Genes	Group of Genes	Names of Genes	Number
Photosynthesis	Subunits of photosystem I	*psaA psaB psaC psaI psaJ*	5
	Subunits of photosystem II	*psbA psbB psbC psbD psbE psbF psbH psbI psbJ psbK psbL psbM psbN psbZ psbT*	15
	Subunits of cytochrome b/f complex	petA petB* petD* petG petL petN	6
	Large subunit of rubisco	*rbcL*	1
	Subunits of ATP synthase	atpA atpB atpE atpF* atpH atpI	6
	Subunits of NADH-dehydrogenase	ndhA* ndhB*^a^ ndhC ndhD ndhE ndhF ndhG ndhH ndhI ndhJ ndhK	12
Self-replication	Ribosomal RNA genes	*rrn16^a^ rrn23^a^ rrn4.5^a^ rrn5^a^*	8
	Small subunit of ribosome	rps2 rps3 rps4 rps7^a^ rps8 rps11 rps12**^a^ rps14 rps15 rps16* rps18 rps19	14
	Large subunit of ribosome	rpl2*^a^ rpl14 rpl16 rpl20 rpl22 rpl23^a^ rpl32 rpl33 rpl36	11
	Transfer RNA genes	trnH-GUG trnK-UUU* trnQ-UUG trnS-GCU trnG-UCC trnR-UCU trnC-GCA trnD-GUC trnY-GUA trnE-UUC trnT-GGU trnS-UGA trnG-GCC* trnfM-CAU trnS-GGA trnT-UGU trnL-UAA* trnF-GAA trnV-UAC* trnM-CAU trnW-CCA trnP-UGG trnI-CAU^a^ trnL-CAA^a^ trnV-GAC^a^ trnI-GAU*^a^ trnA-UGC*^a^ trnR-ACG^a^ trnN-GUU^a^ trnL-UAG	37
	DNA-dependent RNA polymerase	rpoA rpoB rpoC1* rpoC2	4
Other genes	Maturase	*matK*	1
	Envelope membrane protein	*cemA*	1
	Subunit of acetyl-CoA	*accD*	1
	C-type cytochrome synthesis gene	*ccsA*	1
	Protease	clpP**	1
Genes of unknown function	Conserved open reading frames	*ycf1* ^a^ *ycf2* ^a^ ycf3** ycf4 ycf15 ^a^	8

Note: ^a^ Two copies in the IR region; * one intron; ** two introns.

## Data Availability

The genome sequence data that support the findings of this study are openly available in the NCBI GenBank at https://www.ncbi.nlm.nih.gov/nuccore/MW900251, accessed on 23 March 2023, under accession number MW900251. The associated BioProject, SRA, and BioSample numbers are PRJNA721268, SRR14211765 and SAMN18713453.

## Supplementary Materials

The following supporting information can be downloaded at https://www.mdpi.com/article/10.3390/genes15050550/s1 Table S1 Codon preference analysis data. Table S2. Interspersed repeat sequence data and distribution. Table S3. Simple sequence-repeat data and distribution. Table S4. SSR primer sequence list based on the kohlrabi chloroplast genome sequence.
